# Association between fluid overload and SOFA score kinetics in septic shock patients: a retrospective multicenter study

**DOI:** 10.1186/s40560-019-0394-0

**Published:** 2019-08-09

**Authors:** Xavier Chapalain, Véronique Vermeersch, Pierre-Yves Egreteau, Gwenael Prat, Zarrin Alavi, Eric Vicaut, Olivier Huet

**Affiliations:** 1 Department of anesthesiology and intensive care unit, CHRU de Brest Hopital La cavale Blanche, Boulevard Tanguy Prigent, Brest, France; 2 APHP, Unité de recherche clinique, Hôpital Fernand Widal, Université Paris Diderot, Paris, France; 3 Department of Medical ICU, CHRU de Brest, Boulevard Tanguy Prigent, Paris, France; 4 Department of Intensive Care Medicine, CH de Morlaix, Rue de Kersaint Gilly, Morlaix, France; 5 INSERM CIC 1412, CHRU de Brest, Hopital de la Cavale Blanche, Boulevard Tanguy Prigent, Paris, France; 6 ATLANREA Clinical Trial Group, https://www.atlanrea.org/; 7 Université de Bretagne Occidentale, UFR de Médecine, Brest, France

**Keywords:** Sepsis, Septic shock, Fluid therapy, Water-electrolyte balance, Intensive care unit

## Abstract

**Background:**

Fluid infusion represents one of the cornerstones of resuscitation therapies in order to increase oxygen delivery during septic shock. Fluid overload as a consequence of excessive fluid administration seems to be linked to worse long-term outcome. However, its immediate effect on patient’s clinical state is poorly described. The goal of this study was to assess the impact of FO on SOFA score kinetics as a surrogate marker of organ dysfunction from day 0 to day 5.

**Material and methods:**

Retrospective, multicenter, investigator-initiated study. All adult patients (> 18 years old) admitted from January 2012 to April 2017 in one of the three ICUs for septic shock, secondary to peritonitis or pulmonary infection and mechanically ventilated, were included. Univariate analysis was performed with Student’s *t* and chi-square test, for continuous and categorical variables, respectively. A multivariate linear regression model evaluated the impact of FO on delta SOFA score from day 0 to day 5. Secondly, a multivariate mixed-model accounting for repeated measures analyzed the impact of FO on SOFA score kinetics.

**Results:**

One hundred twenty-nine patients met the inclusion criteria and were assigned into FO and no FO groups. FO occurred in 39% of the patients. The difference between SOFA score at day 0 and day 5 was more than twofold higher in the no FO group than in the FO group with a difference of 2.37 between the two groups (4.52 vs. 2.15; *p* = 0.001). Cumulative fluid intake at day 5 was higher in the FO group (2738 vs. 8715 ml, *p* < 0.001). In multivariate analysis, FO was associated with delta SOFA score: aRR = 0.15 (95% CI 0.03–0.63; *p* = 0.009). In mixed model, the regression coefficient for fluid overload status (*r*^*2*^ = 1.16; *p* = 0.014) indicated that the slope for SOFA score kinetic was less pronounced for patients with FO than for patients without FO.

**Conclusions:**

FO patients had a more prolonged multi-organ failure according to SOFA score kinetics during septic shock from resuscitation phase to day 5.

**Electronic supplementary material:**

The online version of this article (10.1186/s40560-019-0394-0) contains supplementary material, which is available to authorized users.

## Background

Sepsis is still a major cause of mortality over the world [1, 2]. A recent systematic review reported an annual incidence of 256 hospital-treated sepsis cases per 100,000 person and per year [3]. It represents nearly 10% of intensive care unit (ICU) admissions, with an average ICU mortality of 18% and a 22% in-hospital mortality regardless of the source of infection [4]. Sepsis management-related cost accounted for 5.2% of total US health cost in 2011 [5].

Distinct phases of hemodynamic resuscitation have been described with different risks, goals, and challenges: resuscitation, optimization, stabilization, and de-escalation phases [6]. Fluid therapy represents one of the cornerstones of resuscitation treatments in order to increase oxygen delivery during circulatory failure [7]. During the salvation phase of septic shock, the current guidelines suggest that an aggressive fluid resuscitation is the best initial therapy [8]. During the optimization phase, the goal is to maintain adequate tissue perfusion and avoid the effects of fluid overload. During this phase, “liberal” or uncontrolled fluid therapy can induce an increased positive fluid balance with tissue fluid overload leading to potential harmful effects [9–12]. A restrictive fluid therapy strategy could be used to decrease fluid overload (FO) during the optimization phase in septic shock patients [13]. It is worth mentioning that inappropriate use of fluid therapy can induce its own side effects.

Therefore, a paradigm shift is currently occurring as concerns have been raised about the potential adverse effects of fluid therapy. Fluid overload is one of the major adverse effects reported and an independent factor of worse outcome in intensive care unit (ICU) patients [10]. It is now suggested that fluid administration should be conducted cautiously to avoid an unnecessary increase in fluid intake but ensure an adequate tissue perfusion. A patient goal-directed therapy may help to optimize fluid intake and avoid the deleterious effects of an increased fluid balance [14].

Most studies on FO or cumulative fluid balance have reported impact on in-hospital or 28-day mortality. To date, there is no data on the impact of FO on organ dysfunction. The aim of this pilot study was to describe the impact of FO on the kinetics of organ dysfunction assessed by the sequential organ failure (SOFA) score between day 0 and day 5 after the onset of septic shock.

## Methods

### Study design and population

We retrospectively analyzed data from three French ICUs at Brest and Morlaix Hospital (France): two units of a teaching hospital (medical ICU and surgical ICU) and one general ICU of a regional hospital. An approval of an institutional ethics committee was obtained before recruitment (approval number: ADZ/Avis no 2017-1). From institutional registries, we identified all adult patients (> 18 years) admitted from January 2012 to April 2017 in one of the three ICUs for septic shock, secondary to peritonitis or pulmonary infection and mechanically ventilated, were eligible for the study. In our cohort, septic shock was defined according to recommendations published in 2003 by Levy et al. [8]. We considered this definition because the more recent Sepsis-3 definition published in 2016 was not already used in daily practice from 2012 to 2017 the participating ICUs [9]. Thus, in our study, septic shock was defined by a suspected or documented infection, clinical/biological signs of systemic inflammatory response syndrome (SIRS), signs of organ dysfunction, and a persistent hypotension (defined as a systolic arterial pressure < 90 mmHg, mean arterial pressure < 65 mmHg, or a reduction in systolic arterial pressure of more than 40 mmHg from baseline) despite fluid challenge needed vasopressors. Diagnosis criteria of intra-abdominal and pulmonary infection used were the one reported in the most recent international guidelines [15–17]:

- Intra-abdominal infection was suspected when the following criteria were met: digestive symptoms (acute abdominal pain, nausea, vomiting, anorexia), acute abdominal contracture or splinting, diffuse abdominal rigidity, and/or hyperthermia > 38.5 °C or hypothermia < 36 °C. Clinical suspicion was systematically confirmed by abdominal CT scan. Then, diagnostic laparoscopy confirmed peritonitis. Peritoneal fluid/tissue was systematically collected from the site of infection for microbiological analysis to identify pathogens [16]
- Diagnosis of pulmonary infection was made using the following clinical criteria: presence of respiratory symptoms (cough, sputum, dyspnea, thoracic pain), hyperthermia > 38.5 °C or hypothermia < 36 °C, and signs of infection on chest radiography. Respiratory samples (sputum, endotracheal aspiration, or bronchoalveolar lavage) were performed to confirm diagnosis and identify pathogens

Patients included in our cohort required mechanical ventilation because they had:

- An acute circulatory failure (defined by systolic arterial pressure < 90 mmHg or mean arterial pressure < 65 mmHg despite fluid challenge and needing of vasopressors > 0.3 μg/kg/min)
- An alteration of consciousness (defined by a Glasgow Coma Scale < 8)
- And/or an acute respiratory failure (defined by a respiratory rate > 35/min, SpO2 < 92% despite non-invasive support and signs of dyspnea)

Patients admitted for septic shock without catecholamine and/or mechanical ventilation were excluded. Patients firstly admitted for another reason were also excluded, even if they presented septic shock during their stay in ICU.

### Data collection

All data were collected from medical records. For eligible patients, the following data were recorded: age, reason for ICU admission, source of infection, hemodynamic support, and respiratory devices used. For enrolled patients, demographic data, baseline body weight, and comorbidities were collected. We evaluated baseline severity with SOFA score and APACHE II score. Initial hemodynamic status (mean blood pressure, heart rate) and hemodynamic treatment received prior to admission (fluid challenge, amount of each catecholamine received) were also recorded. Relevant biological data were collected, especially data needed to obtain SOFA score and lactatemia.

Daily fluid intake from day 0 to day 10 (including fluid challenge, maintenance fluid, and nutrition), daily fluid output (including urine output, insensible losses, drain fluid, ultrafiltration rate, and estimated gastrointestinal losses), and daily fluid balance from day 1 to day 5 (calculated by subtracting the daily fluid output from daily fluid intake) were collected. Daily body weight was recorded. When the recorded body weight was 10% higher than the baseline one, FO was reported. Duration of FO was also recorded. In case of missing baseline or daily body weight, FO status could not be obtained and was marked as not available. All relevant clinical variables necessary to calculate daily SOFA score were collected. For neurological SOFA sub-score, Glasgow coma scale (GCS) was calculated with results of clinical examination. If daily GCS or neurological information were not available and if a patient was sedated, we considered the last GCS reported in the medical record. For non-survivors at day 5, delta SOFA score was calculated taking into account the last observation. We also recorded daily organ support (RRT, mechanical ventilation, amount of catecholamine), length of ICU stay, and mortality (28-day and 90-day).

### Study outcomes

The main pre-specified objective of this study was the impact of FO on SOFA score kinetic from day 0 to day 5 following the onset of septic shock. The onset of septic shock was defined by the time of first antibiotic administration. We chose to evaluate SOFA score kinetics instead of other scores like MODS, SAPS II, and APACHE II because a sequential assessment of organ dysfunction with the SOFA score is robust and has been validated in ICU patients [18]. Several definitions of fluid overload (FO) were used. In some studies, FO was defined by dividing cumulative fluid balance (in liters) by patient’s baseline body weight; a cutoff value of 10% of fluid accumulation was used to define FO [19, 20]. Other studies suggested that a 10% increase in body weight is also clinically relevant [21]. In our study, we identified exposition to FO by dividing daily body weight by baseline body weight. A cutoff value of 10% of weight gain associated with peripheral edema was used to define FO. We hypothesized that FO increases the risk of persistent organ dysfunction during the first 5 days of septic shock.

We also analyzed the impact of patient’s baseline variables on SOFA score kinetics and the impact fluid overload on the following outcomes: 28-day and 90-day mortality, length of stay in ICU, number of ventilator-free days at day 28, and number of catecholamine-free days at day 10.

### Statistical analysis

Population description was described as means and standard deviation for continuous variables and percentages for categorical variables. For any variable with less than 20% of data missing, multiple imputation was used with five iterations, except for categorical variables. Bivariate analysis was performed using student *t* test or Wilcoxon test for continuous variables and chi-square or Fisher’s exact test for categorical variables. Variables with significant bivariate relation (*p* < 0.1) to FO status were considered for multivariate analysis.

A multivariate linear regression model was performed to test the impact of FO on delta SOFA score from day 0 to day 5. The results were presented with risk ratio (RR), 95% confidence intervals (CI), and *p* value. Interactions between FO status and all variables were included in the multivariate model if required. Sensitivity analysis was performed: without outliers, without early dead patients (before day 5), and after being discarded to test assumption after bootstrap replication.

A mixed model was used to evaluate the impact of FO and duration of FO (0 to 1 day, 2 to 5 days, or more than 5 days) on SOFA score kinetics. A “subject” random effect was introduced into the model and was regarded as a fixed effect. “Time” variable was introduced into the model and was regarded as a fixed effect. The mixed model was adjusted for variables that were first in multivariate analysis and found significantly associated with delta SOFA score. Results were presented as coefficients and estimated *p* value (with Satterthwaite approximation method). For multivariate analysis, we considered two-tailed *p* values of less than 0.05 as significant. All statistical analysis was performed with R statistical software (version 3.3.2).

## Results

### Study population

From 1 January 2012 to 31 April 2017, 1209 patients were admitted in participating ICUs for severe sepsis or septic shock. During the study period, 275 eligible patients had septic shock with pneumonia or peritonitis. One hundred forty-six patients were excluded, 61 because they were admitted in ICU for another reason and 55 did not require mechanical ventilation. All included patients (129 over 275) were analyzed. Additional file 1: Figure S1 displays the flow of patients in the study.

### Overall population characteristics and outcomes

Baseline characteristics of patients are summarized in Table 1. The first source of infection was pneumonia (75.2%), and the mean age was 65 years old. On ICU admission, the mean lactate level was 4.37 mmol/l (SD = 4.19) and leucocyte count was 13.97 G/l (SD = 10.85). The overall 28-day mortality was 34.1%, and the mean ICU length of stay was 17.15 days (SD = 19.06). About 39% of patients were exposed to FO during their stay in ICU. Percentages of FO exposure according to ICUs are presented in Additional file 2: Figure S2. For nine patients (7%), no weight was recorded during their stay and FO status was not available. Fluid intakes in the whole cohort were 2017 ml (SD = 2612), 13,320 ml (SD = 7018), and 24,307 ml (SD = 11,658) at day 0, day 5, and day 10 respectively. Daily fluid balance was 1,604 ml (SD = 2806) at day 1 and progressively decreased to 1263 ml (SD = 2955) at day 3 and 91 ml (SD = 1325) at day 5. The cumulative fluid balance at day 5 was 5041 ml (SD = 6789). All studied patients needed noradrenaline support during their ICU stay, 32 (25%) were treated with dobutamine, and 19 (14.8%) were treated with adrenaline at least 1 day. At day 1, the mean infusion rate was 0.4 μg/kg/min for noradrenaline and 5.6 μg/kg/min for dobutamine. At day 5, 25 and 5 patients needed noradrenaline and dobutamine infusion respectively. Mean catecholamine-free days at day 10 was 4.5 days (SD = 3.17).

**Table 1:** Characteristics of overall population according to fluid overload status

Characteristics	All subjects, (*n* = 129)	No fluid overload, (*n* = 73)	Fluid overload, (*n* = 47)	*p* value
Age, years, mean (SD)	65.7 (12)	66.9 (10)	63.2 (13)	0.091^§^
Weight at baseline, kg, mean (SD)	77.3 (19.6)	80 (20.6)	72.3 (17)	0.035^§^
Site of infection, *n* (%)				
Pneumonia	94 (75.2)	55 (78.6)	32 (69.6)	0.381
Peritonitis	32 (25.6)	16 (22.9)	14 (30.4)	0.487
Comorbidities, *n* (%)				
Immunosuppression	32 (25.4)	19 (26.4)	11 (23.9)	0.933
Cardio vascular disease	65 (51.6)	43 (59.7)	16 (34.8)	0.014^§^
Neurological disease	27 (21.4)	14 (19.4)	11 (23.9)	0.728
Psychiatric disorder	24 (19.0)	12 (16.7)	11 (23.9)	0.465
Neoplasm	37 (29.4)	19 (26.4)	17 (37.0)	0.312
Chronic respiratory disease	41 (32.5)	26 (36.1)	12 (26.1)	0.350
Chronic liver failure	23 (18.3)	14 (19.4)	8 (17.4)	0.971
Metabolic disorder	36 (28.6)	23 (31.9)	10 (21.7)	0.320
Chronic renal insufficiency	19 (15.1)	5 (6.9)	9 (19.6)	0.076^§^
SOFA score at baseline, mean (SD)	8.90 (2.99)	8.60 (3.15)	9.09 (2.72)	0.390
APACHE II at baseline, mean (SD)	25.28 (4.84)	25.3 (4.85)	25.1 (4.93)	0.88
Daily FB, ml, mean (SD)				
Day 1	1604 (2806)	1163 (1848)	2117 (3915)	0.075^§^
Day 2	1618 (1547)	1142 (1431)	2387 (1443)	< 0.001^§^
Day 3	1263 (2955)	608 (1387)	2475 (4213)	0.001^§^
Day 4	459 (1642)	88 (1561)	1041 (1653)	0.002^§^
Day 5	91 (1325)	− 272 (1184)	691 (1410)	< 0.001^§^
Cumulative FB from day 1 to day 5, mL, mean (SD)	5041 (6789)	2738 (4217)	8715 (8619)	< 0.001^§^
Delta SOFA score from day 0 to day 5, mean (SD)	3.55 (4.01)	4.52 (3.74)	2.15 (3.50)	0.001^§^
Outcomes				
ICU length of stay, mean (SD)	17.15 (19.06)	11.71 (10.61)	27.66 (25.35)	< 0.001^§^
28-day mortality, *n* (%)	44 (34.1)	21 (28.8)	15 (31.9)	0.870
90-day mortality, *n* (%)	55 (42.6)	25 (34.2)	22 (46.8)	0.236
Ventilator free at day 28, mean (SD)	10.81 (10.59)	13.84 (10.96)	7.19 (8.43)	0.001^§^
Duration of RRT, mean (SD)	4.50 (3.17)	5.21 (3.18)	3.85 (2.83)	0.019^§^
Catecholamine free at day 10, mean (SD)	1.77 (4.47)	1.47 (4.92)	2.45 (4.04)	0.257

*FB* fluid balance, *SD* standard deviation, *RRT* renal replacement therapy

^§^*p* values considered statistically significant

### Between-group differences according to fluid overload status

**Fig. 1:**
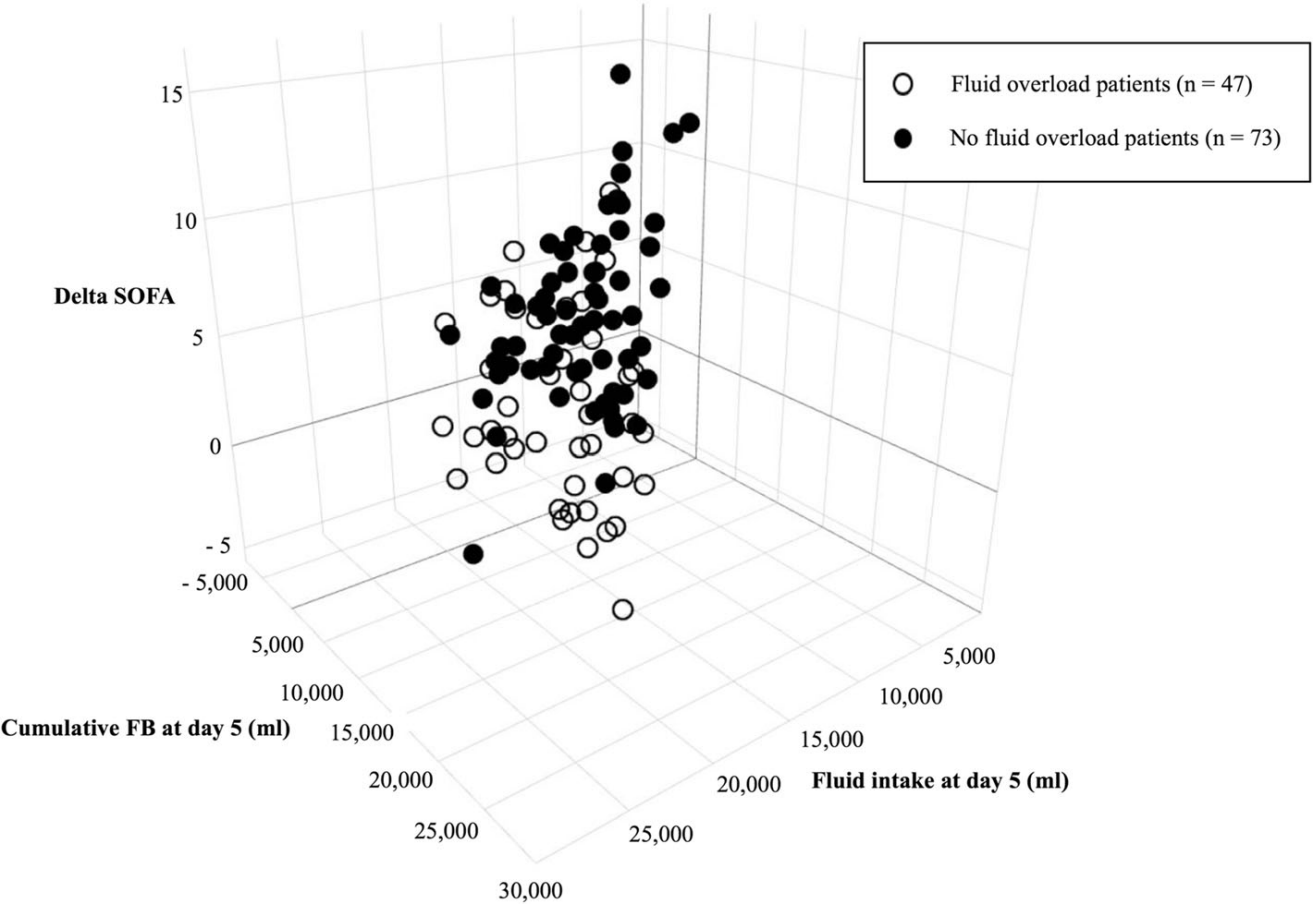
Delta SOFA score, fluid intake, and cumulative FB at day 5 according to FO status. Delta SOFA score from day 0 to day 5 was higher in the group of patients without FO compared to the group of patients with FO (mean delta SOFA score: 4.52 (+/− 3.74) vs. 2.15 (+/− 3.50), *p* < 0.001). Fluid intake at day 5 was more important in the FO group, with a between-group difference of 5709 ml (11,171 ml vs. 16,880 ml, *p* < 0.001). Cumulative fluid balance at day 5 was more important in the FO group, with a between-group difference of 5977 ml (2738 vs. 8715 ml, *p* < 0.001)

Patient’s characteristics according to fluid overload status are summarized in Table 1. Patients exposed to FO had more cardiovascular comorbidities than the non-exposed patients (59.7% vs. 34.8%, *p* = 0.014). At baseline, there was no difference in terms of leucocyte count and lactate level between the two groups. Baseline SOFA score (8.60 vs. 9.09, *p* = 0.39) and APACHE II score (25.3 vs. 25.1, *p* = 0.88) were not statistically different between the two groups. A number of patients treated with dobutamine (23.3% vs. 25.5%, *p* = 0.95) and adrenaline (15.1% vs. 12.8%, *p* = 0.932) were comparable between the two groups. The amount of norepinephrine infused was higher in patients with FO; however, this difference did not reach statistical significance. There was no difference in terms of dobutamine infusion. Fluid intake differences were respectively 1223 ml (*p* = 0.014), 5709 ml (*p* <  0.001), and 11,719 ml (*p* <  0.001) at day 0, day 5, and day 10. Daily fluid balance differences were respectively 954 ml (*p* = 0.075), 1245 ml (*p* <  0.001), 1867 ml (*p* = 0.001), 953 ml (*p* = 0.002), and 963 ml (*p* <  0.001) from day 1 to day 5. Cumulative fluid balance at day 5 was also more important in the FO group, with a between-group difference of 5977 ml (2738 versus 8715 ml, *p* < 0.001). Distribution of cumulative fluid balance and fluid intake at day 5 according to fluid overload status is represented in Fig. 1. There were more transfusions in the FO group (*p* = 0.007). Baseline measurement of SOFA score was similar in two groups (8.60 versus 9.09, *p* = 0.390)*.*

Concerning clinical outcomes, patients without FO were more rapidly discharged from ICU compared to patients with FO with a between-group difference of 6 days (*p* < 0.001). The mean duration of mechanical ventilation was less important for patients without FO than FO patients (13.84 days vs. 7.19 days, *p* = 0.001). The mean duration of RRT was more important in patients without FO (5.21 days versus 3.85, *p* = 0.019). No between-group difference in catecholamine free-days was found. There was no statistically significant difference between groups in mortality at 28 days and 90 days. Bivariate analysis results are displayed in Table 1.

### Daily SOFA score kinetics according to fluid overload status

The difference between SOFA score at day 0 and day 5 (delta SOFA score) was more than twofold higher in the no FO group than in the FO group with a difference of 2.37 between the two groups (*p* = 0.001). Delta SOFA score was also significantly different between the two groups at day 3 (no FO 2.52 +/− 3.39 vs FO 1.11 +/− 3.47; *p* = 0.034) and day 4 (no FO 3.74 +/− 3.39 vs FO 1.72 +/− 3.49; *p* = 0.003). Considering daily measurement of the SOFA score, the main differences occurred from day 3, with respectively a difference of 2.03 (*p* = 0.002), 2.69 (*p* < 0.001), and 2.83 (*p* < 0.001) in day 3, day 4, and day 5. Distribution of delta SOFA score according to fluid overload status is represented in Fig. 1. The mean daily SOFA score from day 0 to day 5 is presented in Additional file 3: Table S1.

**Fig. 2:**
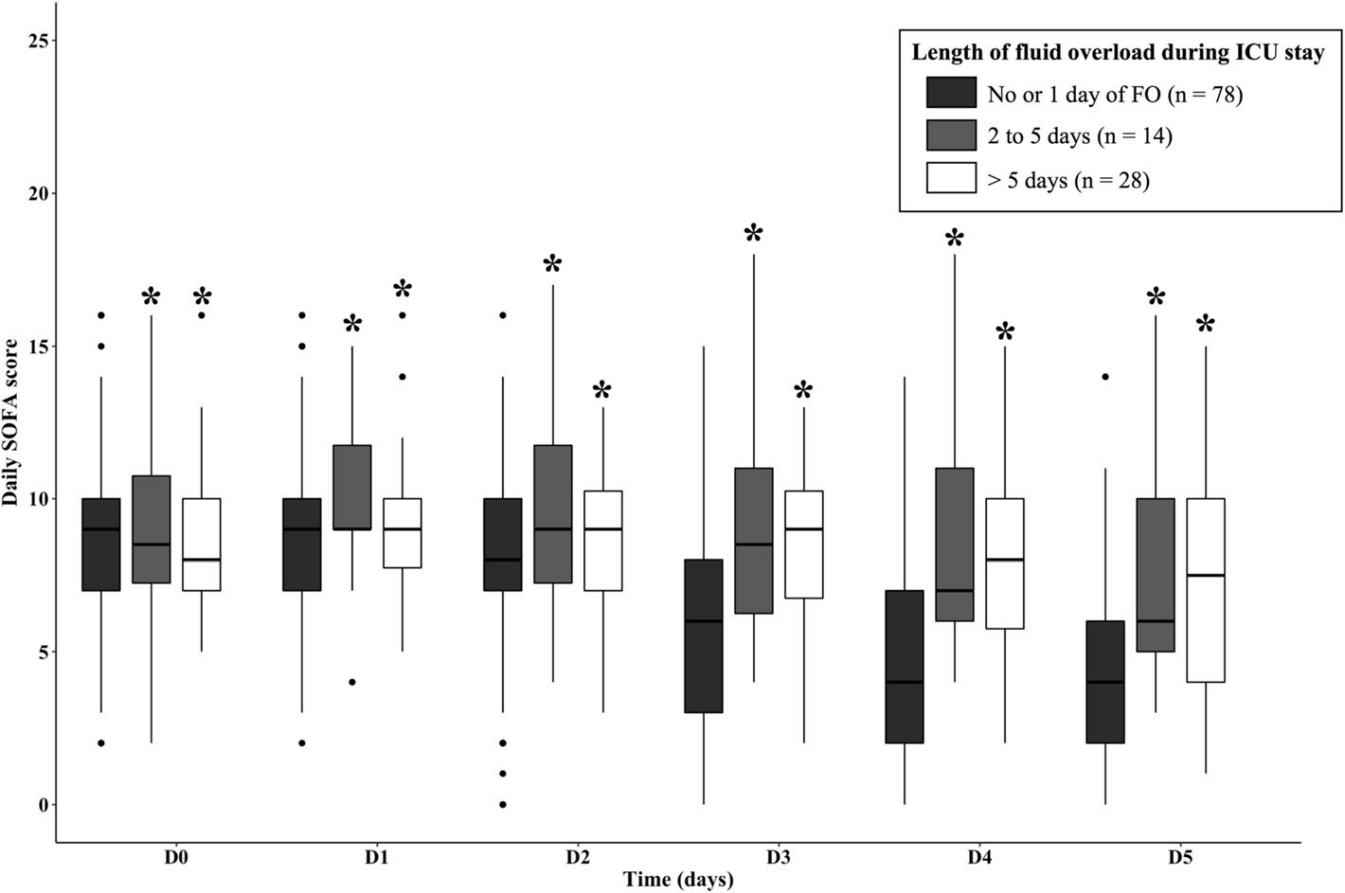
Daily SOFA score from day 0 to day 5 according to the length of FO. Linear mixed model was performed to identify any differences at each time between three sub-groups: No fluid overload or 1 day of fluid overload, 2 to 5 days of fluid overload, more than 5 days. Regression coefficient for the length of FO of 2.1 (*p* = 0.008) for patients with 2 to 5 days of FO and 1.4 (*p* = 0.012) for patients with more than 5 days of FO. These results indicated that the slope for SOFA score kinetics was less pronounced for patients with more than 2 days of FO compared to patients without FO or 1 day of FO (*p < 0.05)

We also analyzed the changes in daily SOFA score according to the length of FO. These results are presented in Fig. 2. There was an association between length of fluid overload and daily SOFA score from day 3 to day 5 (*p* < 0.001). The delta SOFA score was also higher in the group of patients without FO or 1 day of FO compared to the group of patients with 2 or more days of FO (*p* < 0.001).

### Impact of fluid overload on SOFA score kinetics

#### Fixed effect model analysis

A multivariate analysis was used to evaluate the impact of fluid overload on delta SOFA score. The independent variables which were imbalanced between FO and no FO patients were tested in the fixed effect model: FO, age, weight at admission, cardiovascular disease, chronic renal insufficiency, fluid intake at baseline, SOFA score at baseline, heart rate at baseline, and length of hydrocortisone infusion. In the unadjusted analysis, fluid overload status was significantly associated with changes in delta SOFA score (RR = 0.09; 95% CI 0.023–0.38, *p* = 0.001). The other variables significantly associated with delta SOFA score were age (*p* = 0.006), preexisting cardiovascular disease (*p* = 0.011), and SOFA score at baseline (*p* < 0.001). After adjustment, the association between FO status and delta SOFA score persisted (*p* = 0.009). Results of multivariate analysis are presented in Table 2. All sensitivity analysis confirmed this result (details are presented in Additional file 4: Table S2, Additional file 5: Table S3).

**Table 2:** Results of multivariate linear regression model with delta SOFA score as outcome and fluid overload as principal independent covariate

Variables	Unadjusted RR	95% CI	*p* value	Adjusted RR	95% CI	*p* value
Fluid overload status (yes/no)	0.09	[0.023–0.38]	0.001	0.15	[0.03–0.63]	0.009
Covariates						
Age	1.09	[1.02–1.16]	0.006	1.03	[0.97–1.09]	0.4
Weight at baseline	1.00	[0.97–1.04]	0.8	0.98	[0.95–1.01]	0.18
Cardiovascular disease (yes/no)	6.6	[1.55–28.5]	0.011	4.4	[1.10–17.6]	0.036
Chronic renal insufficiency (yes/no)	0.81	[0.10–6.69]	0.84	0.16	[0.02–1.28]	0.084
Fluid intake at baseline	1.0	[0.99–1.00006]	0.122	1.0	[0.9997–1.0002]	0.68
SOFA score at baseline	1.8	[1.43–2.24]	< 0.001	1.72	[1.39–2.13]	< 0.001
Heart rate at baseline	1.0	[0.97–1.29]	0.83	1.0	[0.97–1.03]	0.998
Length of hydrocortisone infusion	0.89	[0.73–1.07]	0.22	0.93	[0.78–1.1]	0.38

*RR* relative risk, *CI* confidence interval

#### Linear mixed model with random effect

Variables associated with delta SOFA score in the adjusted analysis were analyzed in the mixed model after dichotomization (baseline SOFA score < or > 8, fluid overload or not, cardiovascular disease or not). The regression coefficient for FO status of 1.16 (*p* = 0.014) indicated that the slope for SOFA score kinetics was less pronounced for FO patients than for patients without FO whatever the baseline SOFA score was and preexisting cardiovascular comorbidity. The results of the mixed model are presented in Table 3.

**Table 3:** Results of mixed-model analysis with SOFA score as main outcome

Variables	Daily SOFA score					Delta SOFA	Coefficients	*p* value
	Day 1	Day 2	Day 3	Day 4	Day 5			
Fluid overload status								
Yes (*n* = 47)	9.30 (2.53)	8.83 (2.69)	7.98 (3.60)	7.41 (3.72)	7.13 (3.69)	2.15 (3.50)	1.16	0.014
No (*n* = 73)	8.85 (2.84)	7.91 (3.20)	5.95 (3.06)	4.72 (3.14)	4.30 (3.12)	4.52 (3.74)		
Baseline SOFA score								
< 8 (*n* = 60)	7.53 (1.88)	7.09 (2.52)	6.16 (2.79)	5.19 (3.21)	4.54 (3.14)	2.04 (3.28)	2.50	< 0.001
> 8 (*n* = 68)	10.52 (2.61)	9.40 (3.10)	7.53 (3.83)	6.64 (3.95)	6.36 (3.91)	4.92 (4.14)		
Cardiovascular disease								
Yes (*n* = 65)	8.92 (2.86)	7.98 (3.01)	6.15 (3.45)	5.09 (3.77)	4.61 (3.68)	4.53 (4.44)	− 0.60	0.183
No (*n* = 61)	9.25 (2.62)	8.49 (3.10)	7.45 (3.33)	6.65 (3.43)	6.20 (3.52)	2.63 (3.37)		

## Discussion

Our study investigates the association between FO and SOFA score kinetics in septic shock. The main findings of this study are as follows: (1) 40% of our septic shock patients experienced FO; (2) FO patients presented a more prolonged multi-organ failure during septic shock from resuscitation phase to day 5; and (3) the longer the duration of FO, the longer the duration of multi-organ failure.

Fluid therapy and the use of vasopressors are the cornerstones for hemodynamic management from the salvation phase to the de-escalation phase during septic shock [6]. After the resuscitation phase, the optimization phase should be characterized by a cautious titration of fluid administration with a serial reassessment of hemodynamic status [22]. “Liberal” fluid management has been shown to be deleterious for ICU and surgical patients [9, 23, 24]. The SOAP (Sepsis Occurrence in Acute ill Patients) study demonstrated that a positive fluid balance was associated with an increased mortality in patients with acute lung injury and acute renal failure whatever baseline severity and comorbidities were [23, 24]. Even if causality between fluid overload and mortality was not strictly proved, many studies confirmed the statistical association between positive fluid balance and mortality and reinforced this hypothesis [11, 12, 25–28]. In our study, there was no association between FO and 28-day mortality in the bivariate analysis. There is a trend of an increase of 90-day mortality in FO patients, but this result is not statistically significant. However, our study was not designed to test this hypothesis and therefore lack of statistical power.

To our knowledge, our study is the first report demonstrating FO early influence on multiple organ failure kinetic (measured with daily SOFA score). In our cohort, no therapeutic intervention other than fluid management was different between the two groups. In a prospective cohort, Sakr and colleagues identified an association between higher fluid balance (measured at 72 h) and higher mean/maximum SOFA score; however, the authors did not evaluate dynamic SOFA score evolution [11]. In our cohort, a length of fluid overload beyond 2 days seems to be an important determinant of the SOFA score kinetic as the longer FO duration, the longer lasting organ dysfunction. Serial SOFA score measurement is clinically meaningful and reflects global patient’s deterioration or improvement during the course of sepsis. Such association reinforced the link between FO and morbidity during sepsis. These findings are of interest as designing sepsis trial based on non-fatal outcomes (as short-term organ dysfunction) was recently encouraged by experts position paper [29].

In our study, given the longer ventilator-free days and shorter ICU stay, patients without FO had better short-term outcomes. These results were in line with several studies [30–33]. In a recent meta-analysis, considering a mixed population of septic and ARDS patients, Silversides et al. demonstrated that patients included in “liberal” fluid management group had higher mortality, length of stay in ICU, and length of mechanical ventilation [34]. The FACCT study tested in ARDS patients two fluid management strategies [30]. This study found a benefit in terms of ventilator free-days and ICU stay for patients included in the “conservative” strategy. On the other hand, compared to patients without FO, length of RRT was more important in the FO group despite a better SOFA score kinetic (*p* = 0.019). We hypothesized that the use of prolonged dialysis session helped clinicians to avoid FO in maintaining zero or negative net fluid balance. We cannot confirm this hypothesis because we did not collect specifically renal SOFA score and daily diuresis.

To our knowledge, only two pilot studies concluded that a protocolized fluid management can reduce fluid administration without harmful effects during septic shock [31, 32]. In the CLASSIC trial, Hjortrup et al. investigated a fluid restriction protocol compared to the standard care group [31]. Patients included in the interventional group received 250–500 ml of crystalloid boluses in case of severe hypoperfusion. In the standard group, patients received fluid as long as hemodynamic indices improved. Patients included in the fluid “restrictive” group received significantly less fluid during the first 5 days with a mean difference of − 1241 ml. Cumulative fluid balance was also less important: − 1148 ml. There were no differences in the 90-day mortality, but more patients had a new or worsening AKI and ischemic events in the “standard care” group [31]. Chen et al. evaluated a targeted fluid minimization protocol based on passive leg raising. Cumulative fluid balance at day 5 was less important in the interventional group. There were no statistically significant differences in the duration of mechanical ventilation, maximal dose of vasopressor, or in-hospital mortality [32].

Considering the target population of our study, we followed the recommendation underlined by a recent review which criticized the past design of trials focusing on septic patients [29]. In this review, Mebazaa et al. emphasized that designing sepsis trials without considering baseline risk resulted in lower event rates than expected and decreased statistical power. Therefore, we excluded low-risk patients (urosepsis, skin/soft tissue infection) and chose to enroll a homogeneous group of patients with a reported mortality and organ dysfunction consistent for between patients [35].

We acknowledge some limitations of this study. We chose to include septic shock patients according to the sepsis-2 definition because we considered that the more recent Sepsis-3 definition could not be implemented in daily practice in participating ICUs. This is a limitation of our cohort. Then, including only mechanically ventilated patients limits the external validity. Further studies are needed to confirm these results in patients without mechanical ventilation. Therefore, being observational and retrospective, our study results cannot be conclusive in regard to the correlation between FO and SOFA score kinetic. Then, the relatively small number of patients lowers the statistical power of the study. Finally, early death induced missing data in the collection of daily SOFA score. However, we performed a mixed modeling showing the same results, and a sensitivity analysis without missing data was performed. Results were consistent throughout the entire analysis.

## Conclusions

This multicenter retrospective study is the first report of SOFA score kinetics determinants during septic shock. Fluid overload seems to be an independent determinant of SOFA score kinetic. Multi-organ dysfunction is more prolonged for patients with FO compare to patients without FO. This is a hypothesis-generating study; therefore, the results need further studies to be confirmed. Well-designed clinical trials aiming to demonstrate the benefit of using a targeted, pragmatic, and individualized hemodynamic management during the optimization phase are still needed to translate evidences into clinical practice.

## Additional files

Additional file 1: **Figure S1.** Flow chart of the study. (PDF 47 kb)

Additional file 2: **Figure S2.** Number of fluid overloaded patients in the three ICUs. (PDF 52 kb)

Additional file 3: **Table S1.** Daily SOFA score evolution according to fluid overload status. (DOCX 47 kb)

Additional file 4: **Table S2.** Results of multivariate linear regression model with delta SOFA score as outcome and fluid overload as principal independent covariate without early dead patients (*n* = 109). (DOCX 43 kb)

Additional file 5: **Table S3.** Results of multivariate linear regression model with delta SOFA score as outcome and fluid overload as principal independent covariate without influential observations (*n* = 125). (DOCX 48 kb)

## Data Availability

The dataset supporting the conclusions of this article is fully available. To have an access on it, please contact the corresponding author (O.H.)

## Abbreviations

aRR: Adjusted relative risk
CI: Confidence interval
FB: Fluid balance
FO: Fluid overload
ICU: Intensive care unit
RR: Relative risk
RRT: Renal replacement therapy
SOFA: Sequential organ failure assessment
