# Supplementary material for: Association between fluid overload and SOFA score kinetics in septic shock patients: a retrospective multicenter study
Source: J Intensive Care. 2019 Aug 9;7:42. doi: 10.1186/s40560-019-0394-0 (PMC6688320; doi:10.1186/s40560-019-0394-0)
Supplement: Supplementary file 1 — Figure S1. Flow chart of the study. (PDF 47 kb) [file 40560_2019_394_MOESM1_ESM.pdf]

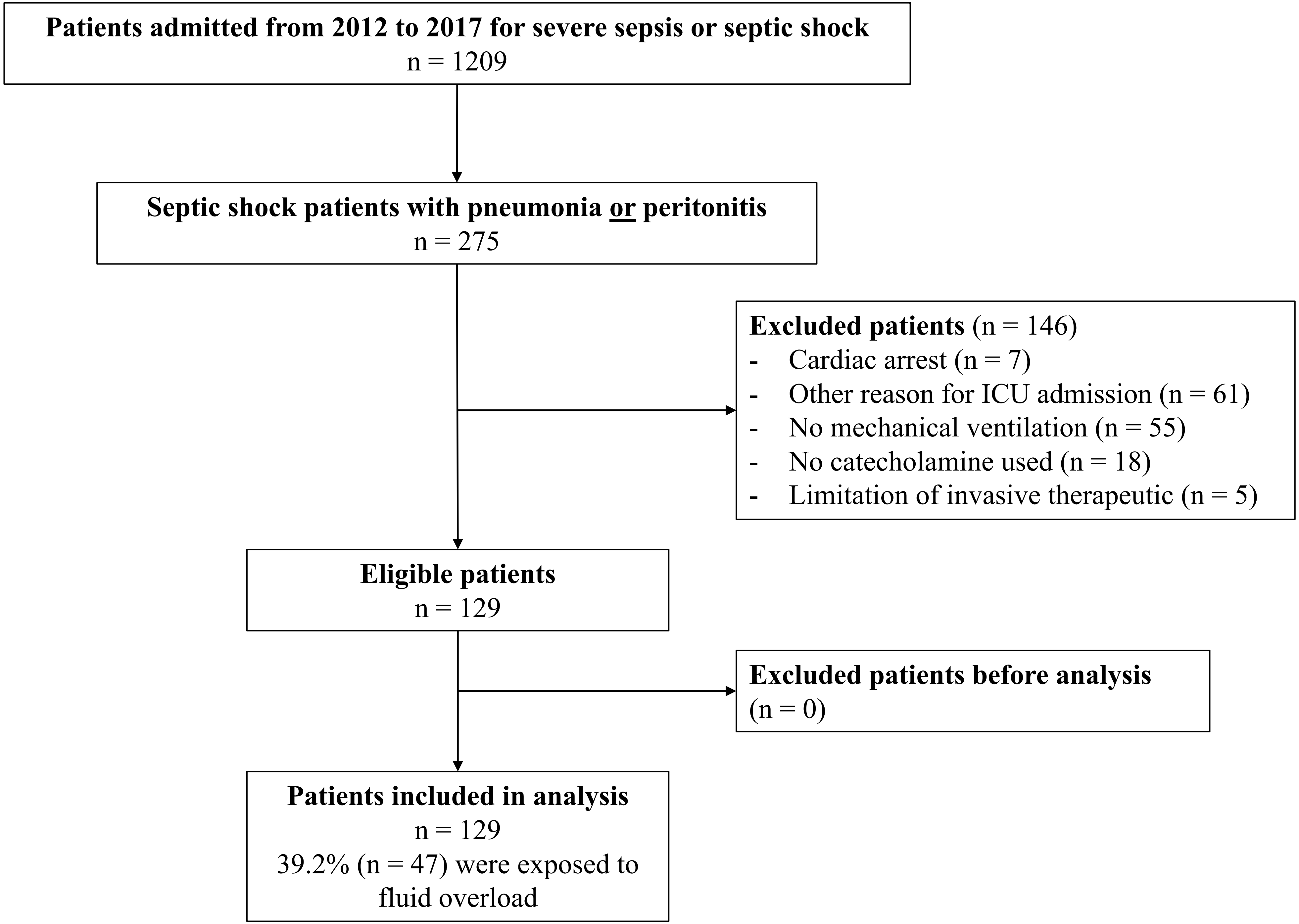
Patients admitted from 2012 to 2017 for severe sepsis or septic shock
n = 1209
Septic shock patients with pneumonia or peritonitis
n = 275
Excluded patients (n = 146)
- Cardiac arrest (n = 7)
- Other reason for ICU admission (n = 61)
- No mechanical ventilation (n = 55)
- No catecholamine used (n = 18)
- Limitation of invasive therapeutic (n = 5)
Eligible patients
n = 129
Excluded patients before analysis
(n = 0)
Patients included in analysis
n = 129
39.2% (n = 47) were exposed to
fluid overload
