# Supplementary figures and images for: Association between fluid overload and SOFA score kinetics in septic shock patients: a retrospective multicenter study
Source: J Intensive Care. 2019 Aug 9;7:42. doi: 10.1186/s40560-019-0394-0 (PMC6688320; doi:10.1186/s40560-019-0394-0)

**Number of patients**

80  
60  
40  
20  
0

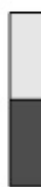

ICU n°1

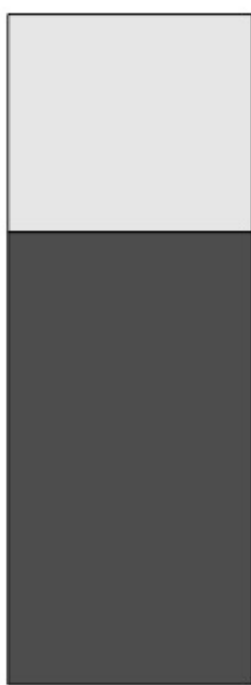

ICU n°2

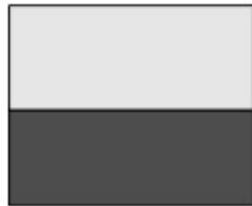

ICU n°3

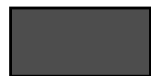

No fluid overload

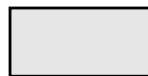

Fluid overload

Supplement: Supplementary file 2 — Figure S2. Number of fluid overloaded patients in the three ICUs. (PDF 52 kb) [file 40560_2019_394_MOESM2_ESM.pdf]
