# Supplementary material for: Association between fluid overload and SOFA score kinetics in septic shock patients: a retrospective multicenter study
Source: J Intensive Care. 2019 Aug 9;7:42. doi: 10.1186/s40560-019-0394-0 (PMC6688320; doi:10.1186/s40560-019-0394-0)
Supplement: Supplementary file 3 — Table S1. Daily SOFA score evolution according to fluid overload status. (DOCX 47 kb) [file 40560_2019_394_MOESM3_ESM.docx]

**Table S1:** Daily SOFA score evolution according to fluid overload status.

| **Variables** | **All subjects, (n = 129)** | **No fluid overload, (n = 73)** | **Fluid overload, (n = 47)** | **P-value** |
| --- | --- | --- | --- | --- |
| **Daily SOFA score, mean +/- SD** |  |  |  |  |
| Day 0 | 8.90 (2.99) | 8.60 (3.15) | 9.09 (2.72) | 0.390 |
| Day 1 | 9.11 (2.72) | 8.85 (2.84) | 9.30 (2.53) | 0.379 |
| Day 2 | 8.28 (3.04) | 7.91 (3.20) | 8.83 (2.69) | 0.110 |
| Day 3 | 6.83 (3.43) | 5.95 (3.06) | 7.98 (3.60) | **0.002*^§^*** |
| Day 4 | 5.89 (3.70) | 4.72 (3.14) | 7.41 (3.72) | **<0.001*^§^*** |
| Day 5 | 5.43 (3.68) | 4.30 (3.12) | 7.13 (3.69) | **<0.001*^§^*** |
|  |  |  |  |  |
| **Delta SOFA score from day 0 to day 5, mean +/- SD** | 3.55 (4.01) | 4.52 (3.74) | 2.15 (3.50) | **0.001*^§^*** |

*^§^* p-values < 0.05.
