# Supplementary material for: Association between fluid overload and SOFA score kinetics in septic shock patients: a retrospective multicenter study
Source: J Intensive Care. 2019 Aug 9;7:42. doi: 10.1186/s40560-019-0394-0 (PMC6688320; doi:10.1186/s40560-019-0394-0)
Supplement: Supplementary file 4 — Table S2. Results of multivariate linear regression model with delta SOFA score as outcome and fluid overload as principal independent covariate without early dead patients (n = 109). (DOCX 43 kb) [file 40560_2019_394_MOESM4_ESM.docx]

**Table S2:** Results of multivariate linear regression model with delta SOFA score as outcome and fluid overload as principal independent covariate without early dead patients (n= 109).

| **Variables** | **Adjusted RR** | **P-value** |
| --- | --- | --- |
| **Fluid overload (Yes/No)** | **0.14** | **0.006** |
|  |  |  |
| **Covariates** |  |  |
| Age | 1.027 | 0.34 |
| Weight at baseline | 0.98 | 0.16 |
| Cardio vascular disease (Yes/No) | 4.18 | 0.04 |
| Chronic renal insufficiency (Yes/No) | 0.19 | 0.11 |
| Fluid intake at baseline | 1.0 | 0.76 |
| SOFA score | 1.68 | < 0.001 |
| Heart rate at baseline | 1.0 | 0.99 |
| Length of hydrocortisone infusion | 0.94 | 0.48 |
|  |  |  |

RR: relative risk.
