# Supplementary material for: Association between fluid overload and SOFA score kinetics in septic shock patients: a retrospective multicenter study
Source: J Intensive Care. 2019 Aug 9;7:42. doi: 10.1186/s40560-019-0394-0 (PMC6688320; doi:10.1186/s40560-019-0394-0)
Supplement: Supplementary file 5 — Table S3. Results of multivariate linear regression model with delta SOFA score as outcome and fluid overload as principal independent covariate without influential observations (n = 125). (DOCX 48 kb) [file 40560_2019_394_MOESM5_ESM.docx]

**Table S3:** Results of multivariate linear regression model with delta SOFA score as outcome and fluid overload as principal independent covariate without influential observations (n = 125)

| **Variables** | **Adjusted RR** | **IC 95%** | **P-value** |
| --- | --- | --- | --- |
| **Fluid overload (Yes/No)** | **0.15** | **[0.038 - 0.56]** | **0.005** |
|  |  |  |  |
| **Covariates** |  |  |  |
| Age | 1.01 | [0.96 - 1.07] | 0.71 |
| Weight at baseline | 0.98 | [0.95 - 1.01] | 0.31 |
| Cardio vascular disease (Yes/No) | 6.42 | [1.7 - 23.1] | 0.006 |
| Chronic renal insufficiency (Yes/No) | 0.066 | [0.009-0.49] | 0.009 |
| Fluid intake at baseline | 0.9998 | [0.9993 - 0.9998] | 0.28 |
| SOFA score | 1.90 | [1.55 - 2.34] | < 0.001 |
| Heart rate at baseline | 1.005 | [0.98 - 1.03] | 0.71 |
| Length of hydrocortisone infusion | 0.95 | [0.80 - 1.11] | 0.49 |
|  |  |  |  |

RR: relative risk, CI: Confidence Interval.
